# Characterization of Bricks from Baroque Monuments in Northeastern Poland: A Comparative Study of Hygric Behavior and Microstructural Properties for Restoration Applications

**DOI:** 10.3390/ma18133023

**Published:** 2025-06-26

**Authors:** Joanna Misiewicz, Maria Tunkiewicz, Gergő Ballai, Ákos Kukovecz

**Affiliations:** 1 Department of Building Engineering and Building Physics, Faculty of Geoengineering, University of Warmia and Mazury in Olsztyn, Jana Heweliusza 4, 10-724 Olsztyn, Poland; joanna.misiewicz@uwm.edu.pl; 2 Department of Applied and Environmental Chemistry, University of Szeged, Rerrich Béla tér 1, H-6720 Szeged, Hungary; ballai.gergo@gmail.com (G.B.); kakos@chem.u-szeged.hu (Á.K.)

**Keywords:** historical buildings, clay brick, hygric properties, compressive strength, freeze–thaw cycles, MIP, XRD, TGA

## Abstract

This study presents a comprehensive material characterization, including physical, hygric, and mechanical properties, of historical ceramic bricks to enhance the understanding of heritage masonry structures and support the effective planning of conservation interventions. The primary objective is to systematize the knowledge of constituent materials in brick walls from different historical periods and to evaluate the compatibility of modern repair materials with the original fabric. To this end, a comprehensive experimental protocol was employed, which included the determination of fundamental physical properties such as density, water absorption, and sorptivity. Additionally, chemical and thermogravimetric analyses were performed, followed by freeze–thaw resistance testing and compressive strength measurements. Microstructural analysis was conducted using mercury intrusion porosimetry. The results identified the pore size ranges most susceptible to frost-induced degradation and revealed correlations between the physical, hygric, and mechanical properties of the tested ceramic materials. These findings provide essential data on the physico-mechanical characteristics of historical bricks, establishing a basis for the informed selection of compatible materials in conservation practice.

## 1. Introduction

The cultural, traditional, and architectural aspects of heritage brick constructions underscore their intrinsic value, which is enhanced by their unique material composition and microstructural features. As a substantial component of national heritage, masonry buildings are the focus of targeted conservation and protection efforts, due to their cultural and historical importance. The International Council on Monuments and Sites (ICOMOS) has established fundamental principles for preservation, recommending that interventions must preserve cultural value and ensure material compatibility [1]. Consequently, any such interventions must be preceded by a comprehensive examination of the original construction techniques and building materials, which have evolved significantly over the centuries.

In Poland, the use of fired brick became widespread during the medieval period and grew in prominence over time, establishing it as an essential building material. While brick dimensions and bonding methods changed over time, the 19th century marked a significant shift with the mechanization of the manufacturing process [2]. In the 20th century, traditional brick was increasingly superseded by modern materials like aerated concrete and silicates, and eventually by the widespread adoption of concrete structures. Despite this evolution, the fundamental production method for fired bricks has remained consistent, generally involving the sourcing of raw materials, mixing with water and occasional additives, shaping, drying, and firing. The final characteristics of the brick are determined by the raw material properties and the specific conditions at each production stage [3,4]. Key factors influencing the physical properties and pore structure include the use of additives (e.g., brick fragments, straw, sand) [5,6,7], the shaping method, and the firing temperature. For instance, historical molding techniques produced greater total porosity compared to modern high-pressure extrusion [8]. Furthermore, historical bricks were typically fired at lower temperatures (<900 °C), resulting in lower density, strength, and durability than modern bricks, which are fired at higher temperatures (>1000 °C) [3,4,9].

Historic brick structures, particularly those designated as monuments, are susceptible to degradation, primarily from exposure to weathering [10,11]. Numerous studies from a materials science perspective identify moisture as a crucial factor in masonry degradation, as the amount of water present directly influences the extent of damage from processes like freeze–thaw cycles, biological corrosion, and salt crystallization. The sorption and capillary transport properties of masonry materials, intrinsically controlled by their pore structure and surface chemical characteristics, critically influence the progression of degradation mechanisms such as freeze–thaw cycling, salt crystallization, and biological activity, thereby substantially impacting the long-term durability and performance of the structures [12,13,14,15,16]. As building façade parts, bricks are exposed to fluctuating temperature and moisture, which induce microstructural changes that can lead to weathering and frost damage [9]. The protection of historical fabric and extension of the long-term performance of existing structures are possible only through well-considered conservation or reconstruction [17,18,19], which necessitate a comprehensive examination of the historical material characteristics of a given period, as well as its contemporary equivalents.

While the literature is relatively extensive with regard to microstructural and physical characteristics, water-related properties, resistance to environmental conditions, and compressive strength [16,17,18,20,21,22], few studies identify currently available, compatible replacement materials capable of achieving structurally homogeneous repairs [23,24,25]. The selection of suitable replacement materials must therefore be based on a suite of laboratory tests, including frost resistance, compressive strength, and microstructural analysis of porosity and moisture characteristics.

The resistance of clay bricks to freeze–thaw cycles has been widely studied with regard to various properties, such as changes in surface appearance [26,27], compressive strength or the propagation speed of ultrasonic waves through specimens [28], weight [20], and pore structure [29,30]. These studies indicate that freeze–thaw cycles introduce new micropores and cracks, damage brick surfaces, and reduce compressive strength. To ensure the homogeneity of a repaired wall, and acknowledging that historic bricks have already endured numerous freeze–thaw cycles, the selection of replacement materials should be based on an assessment of modern bricks that have also been subjected to frost resistance testing. Such an analysis allows for a more accurate evaluation of post-repair structural performance.

Another significant parameter is the water uptake of clay bricks, which is generally expressed as a time-dependent function of fluid absorption [31,32,33,34,35,36]. Capillary suction is a key property that can be quantified using accessible testing methods, with capillary absorption, sorptivity, and vacuum saturation being the most frequently defined parameters, providing essential insights into moisture dynamics within the porous matrix [25,37]. The relationship between moisture absorption and porosity is well-established in the literature [38,39,40], and pore distribution has a decisive influence on the durability of ceramic materials. According to the International Union of Pure and Applied Chemistry (IUPAC) classification [39], pores are categorized as micropores (<2 nm), mesopores (2–100 nm), and macropores (>100 nm). Each class plays a distinct role in moisture transport: micropores govern sorption capacity, mesopores are central to moisture transport and surface sorption, and macropores primarily function as a transport network, effectively distributing moisture throughout the material [38].

The mechanical properties of brick masonry are closely determined by its constituent materials, with its compressive strength being primarily influenced by the mechanical performance of the masonry units [41]. The mean compressive strengths of masonry units, fb, and mortar, fm, have a direct impact on the calculated masonry compressive strength, fk. However, its evaluation is complicated by high variability in historical production standards and the degree of material deterioration.

The mechanical properties of brick masonry components, which are crucial for understanding the relationship between microstructural attributes and mechanical performance, are widely discussed in the materials science literature [9,16,41,42] and form the basis for assessing the strength of existing brick masonry structures in design processes, despite the broad range of values (from 1.5 to 32 N/mm^2^). However, there is a scarcity of information in terms of compressive strength values specific to different historical periods as well as about compressive strength values of modern replacement materials used in restoration processes in relation to historical bricks.

This article is a continuation of the authors’ research presented in [38,43] on clay bricks from various historical periods, as well as modern restoration materials, aimed at ensuring the best possible homogeneity of masonry and the effectiveness of planned repairs through an integrative materials science approach. Here, the authors pursue three main research objectives:

1. To conduct a detailed characterization of the physical, hygric, microstructural, and mechanical properties of Baroque bricks from the 18th and 19th centuries.
2. To assess the compatibility between modern restoration bricks and their historical counterparts based on key technical parameters, including porosity, water absorption, compressive strength, and freeze–thaw resistance.
3. To develop preliminary guidelines for selecting contemporary restoration materials that align with conservation requirements.

To achieve the above objectives, a detailed characterization of both historical and contemporary bricks was carried out in addition to our previously published results on contemporary bricks [43]. The data reported now include the evaluation of frost resistance, mineralogical composition, compressive strength, and changes in pore structure after a defined number of freeze–thaw cycles. In order to determine the aforementioned parameters of the tested samples, various tests were conducted using different techniques (e.g., compressive strength, frost resistance, water sorptivity, capillary absorption coefficient, MIP, XRF, XRD, TGA). The research results enabled comparative analyses focused on assessing the appropriateness of contemporary restoration materials. Furthermore, as the moisture-related and mechanical parameters of porous construction materials—characterized within this materials science framework—constitute essential input parameters for numerical modeling, the results acquired in this study provide a reliable basis for simulations grounded in empirical data. This comprehensive, materials-centric methodology not only deepens the mechanistic understanding of degradation processes in heritage bricks but also establishes a robust scientific foundation for selecting repair materials that ensure long-term structural integrity and the preservation of cultural heritage.

## 2. Materials and Methods

### 2.1. Description of Materials

**Figure 1:**
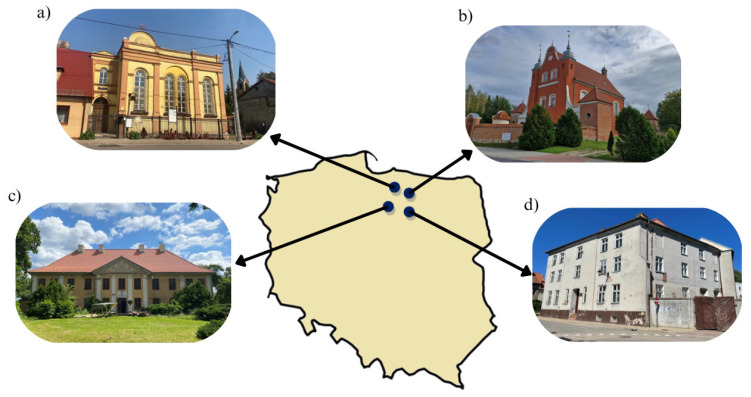
Baroque buildings: (**a**) Bishop’s Palace in Smolajny (54.0281,20.4066), (**b**) Monastery building in Orneta (54.1270,20.1400), (**c**) Church of the Holy Cross and Our Lady of Sorrows in Międzylesie (53.9815,20.4817), (**d**) Synagogue in Barczewo (53.8448,20.5918).

The experimental study utilized brick specimens from four heritage structures in northeastern Poland. The geographical locations of the sites are shown in Figure 1. Samples were carefully extracted during structural restoration processes from sections of the buildings scheduled for demolition.

The Baroque monuments were:

− The Synagogue in Barczewo (Figure 1a): Established in 1847, this two-story, rectangular brick building is the only preserved former synagogue in the Olsztyn district. Its sale to private owners in 1937 contributed to its preservation, as it remained undamaged during Kristallnacht. The structure was constructed in the Neoclassical style, featuring a decorative façade articulated by pilasters and large, semicircular-arched windows.
− The Church of the Holy Cross and Our Lady of Sorrows in Międzylesie (Figure 1b): This Baroque church was constructed from stone and brick between 1752 and 1753 by rebuilding a small 1722 chapel, preserving some of the original walls. Side towers were added in 1755, and a perimeter wall with four corner chapels was completed by 1775. Its distinct features include a triangular bell-shaped gable, a masonry turret for the sanctus bell, and a sundial.
− The Bishop’s Palace in Smolajny (Figure 1c): Situated on a high escarpment along the Łyna River, on the site of a former fortified manor. Constructed between 1741 and 1743 at the behest of Bishop Adam Stanisław Grabowski, the building served as a summer residence for the Bishops of Warmia. Designed in the Baroque style, it is a masonry structure with plastered surfaces, characterized by architectural simplicity enhanced with numerous decorative elements. The palace has a slightly elongated rectangular layout, comprises two stories, and is topped with a hipped roof covered with red ceramic tiles.
− The monastery building in Orneta (Figure 1d): Situated within the defensive walls of the historic town, along the southeastern bend of Olsztyńska Street. The convent of the Sisters of St. Catherine was founded at this location in 1581 by Bishop Marcin Kromer. Archival records from 1565 and 1581 refer to the building as an older structure comprising ten cells. Prior to 1586, it underwent complete reconstruction and was adapted to accommodate the needs of the newly established convent. The edifice is a four-winged masonry structure with plastered facades, arranged around a rectangular internal courtyard. The front wing, on the western side, has walls and cellars dating back to 1586. The remaining wings date from 1776, with the southern wing constructed on the former defensive wall.

To maintain consistent testing conditions and avoid differences due to sample orientation, only bricks from the above-ground portions of perimeter walls were selected, thereby ensuring material uniformity crucial for reliable materials science analyses. The sampling strategy was formulated to systematically account for potential variations in the microstructural and physical properties of the masonry, attributable to differences in environmental exposure and elevation within the structure. Bricks from the Bishop’s Palace in Smolajny were taken from the walls of the ground floor in the central part of the front wall (at a height of approximately 1.80 m above ground level) and from the wall of the first floor located near the southwest corner of the building (at a height of approximately 4.30 m above ground level). Samples from the monastery in Orneta were taken from the northern wall of the southern wing (at a height of approximately 1.50 m above ground level). Bricks from both the church in Międzylesie and the synagogue in Barczewo were taken from the eastern walls. However, the sampling height in the church Międzylesie differs from other buildings as the only available sections of the wall where renovation work was carried out were the attic walls. All specimens were collected from the interior sections of a structurally homogeneous brick masonry wall to minimize environmental and material heterogeneity, ensuring the reliability of subsequent microstructural analyses. Despite the limited availability of material, only samples in good technical condition—free from cracks, major edge or surface damage, and signs of delamination—were qualified for testing. Minor edge chipping was considered acceptable. The selected sample set was deemed representative of the examined material.

Following on from earlier studies of [44] which indicate that modern hand-made bricks are suitable materials for conservation purposes, two types of hand-made bricks were adopted as reference materials due to their material properties relevant from a materials science perspective. The moisture-strength parameters and microstructural characteristics, which are critical in materials science for evaluating the durability and performance of porous building materials, were determined for the aforementioned bricks in [43], and their parameters showed the greatest consistency with the tested historical samples. Modern bricks were produced in manufactories using a hand-molding process based on decades-old methods and, according to the manufacturers’ declarations, are intended for use in historical buildings. In the article, the research on modern bricks was extended to include frost resistance tests as well as strength, sorptivity, water absorption, color definition, mineralogical composition, and porosity after frost resistance testing.

The following brick designations were employed to facilitate clear presentation of the experimental results: for historically manufactured bricks from the Palace in Smolajny, H-SM; from the monastery in Orneta, H-OR; from the church in Międzylesie, H-ML; from the synagogue in Barczewo, H-SB. Contemporary bricks were marked with the symbols M-GO and M-HM—designations consistent with the results of former studies in [43].

### 2.2. Description of Testing Methods

#### 2.2.1. Physical Characterization

Experimental tests were preceded by preparatory work involving the cleaning of brick surfaces from mortar residues. The geometric characterization of historic bricks was performed by determining the effective dimensions of the brick faces, obtaining the length (L), width (W) and height (H) values. The samples were measured with an electronic caliper. Subsequently, the color of all tested bricks was determined using a Konica Minolta CM-700d spectrophotometer (Tokyo, Japan), with the results expressed as color codes in terms of hue, value, and chroma, based on the Munsell color system. While primarily related to aesthetics and heritage value, color measurements also indirectly reflect mineralogical composition and firing conditions, which are critical factors in ceramic material characterization.

Water absorption constitutes a fundamental parameter in materials science, as it quantitatively reflects the porosity, capillary transport capacity, and overall moisture affinity of porous materials, directly influencing their vulnerability to deterioration mechanisms such as freeze–thaw cycling, salt crystallization, and microbial colonization. The study was performed on historic brick samples dried to a constant weight at 105 ± 5 °C, according to the standard PN-EN 772-21:2011 [45]. Dried samples were cooled and placed in water on washers. The samples were positioned vertically on their longest side. The brick samples were soaked in water until the variation between two consecutive measurements was less than 0.2% (every 24 h). The theoretical equation defining the water absorption Ws is the Equation (1):

(1) $$W_{s}=\frac{M_{s}-M_{d}}{M_{d}}\cdot 100\%$$

where *W_s_*—is the water absorption [%], *M_s_*—is the mass of the water-saturated sample [g] and *M_d_*—is the mass of the sample dried to a constant weight [g].

Bulk density measurements were carried out using a Micromeritics AutoPore IV 9500 mercury porosimeter (Micrometrics, Atlanta, GA, USA).

#### 2.2.2. Mineralogical Composition and Thermal Gravimetric Analysis

X-ray diffraction (XRD) measurements were performed using a Rigaku MiniFlex II benchtop diffractometer (Tokyo, Japan) equipped with a Co-Kα radiation source (λ = 1.7902 Å), covering the 5–80° 2θ range at a scan rate of 1° per minute. Since the wavelength of the X-ray source influences the diffraction angle, the 2θ position at which a given diffraction peak appears varies depending on the radiation source used. Conversion to the more widely used Cu Kα radiation source can be performed for each sample in accordance with Equation (2):

(2) $$sin\theta_{Cu}=\frac{\lambda_{Co}}{\lambda_{Cu}}sin\theta_{Co}$$

where *λ*—is the wavelength [Å], *θ*—is the angle of diffraction [°], and λ_Cu_Kα = 1.5406 Å.

Throughout the measurement process the brick samples were left intact and measured with a custom-made, 3D-printed sampleholder. Analysis of the diffraction patterns was conducted using the XPowder computational tool. The chemical composition of the bricks was analyzed using X-ray fluorescence (XRF). The analysis was carried out with a Shimadzu EDX-720 spectrometer (Kyoto, Japan), which irradiates the sample with X-rays and detects the energy of the resulting characteristic fluorescence to identify and quantify the elements present in the specimen. The concentration of oxides in each brick type was determined using XRF analysis, providing essential data on the raw material origin and firing conditions, both of which strongly influence porosity, thermal expansion, and long-term durability.

The weight losses with respect to raising temperature was determined using a TAQ500 instrument (TA Instruments, New Castle, DE, USA) under an air flow, ranging from room temperature to 900 °C, at a heating rate of 10 °C min^−1^. Thermogravimetric analysis data provided detailed insights into the thermal stability, dehydration mechanisms, and decomposition behavior of carbonate phases, which are intrinsically linked to the bricks’ thermal history and their resilience against thermal cycling and environmental degradation.

#### 2.2.3. Microstructure Studies: Microscopy and Mercury Porosimetry Method (MIP)

The microstructure studies began with microscopic measurements aimed at quantitatively characterizing the material morphology and heterogeneity at the meso-scale, crucial for understanding the microstructural factors influencing mechanical and hygric behavior. For the tests, rectangular samples were prepared. The photos were taken in order to present the same plane in each sample. The tests were performed by the use of the Olympus SZX16 microscope (Tokyo, Japan), which enables a system for digital visualization, and the analysis and documentation of images. The measurement set consisted of a stereoscopic microscope connected to a camera and QuickPhoto Camera 2.3 software.

Microstructural studies were also carried out using a mercury porosimeter. MIP is based on the measurement of the volume of mercury introduced into open pores under pressure, which allows the determination of the distribution of cylindrical pores [46]. Measurements of pore characteristics were carried out in accordance with International Standard ISO 15901-1: Evaluation of pore size distribution and porosity of solid materials by mercury porosimetry and gas adsorption—Part 1: Mercury porosimetry. This standard defines the principles for evaluating pore size distribution and porosity of solid materials [47].

Cylindrical brick samples were used for tests using the Autopore IV mercury porosimeter. Each type of brick sample was tested, with a diameter of 12.0 mm and a height of 15.0 mm. Before testing, the samples were dried to a constant mass and cleaned with compressed air. The applied method is based on the principle that mercury progressively penetrates the pore structure under increasing pressure, with the volume of intruded mercury being a function of the applied pressure. The maximum working pressure generated by the device was 228.0 N/mm^2^. The Equation (3) describes the phenomenon related to mercury porosimetry:

(3) $$r_{0}=-\frac{2\gamma_{m}cos\theta_{m}}{p_{m}}$$

where:

$r_{0}$—is the pore radius [nm], $p_{m}$—the pressure [Psi], *γ*—the surface tension of mercury (value assumed was 424 [$\frac{mN}{m}$]), and *θ*—the wetting angle (value assumed was 141.3 [°]).

Using this equation, pore diameters are determined by considering the pressure of the introduced mercury. Mercury porosimetry allows for the acquisition of information regarding pore volume, distribution, average pore diameter, and total pore surface area [48].

#### 2.2.4. Hygric Characterization

Liquid suction is a physical phenomenon, the understanding and quantitative control of which are fundamental in materials science, particularly for porous construction materials, and the control of which is crucial for preventing irreversible destructive processes, as the distribution of moisture in the examined material is closely related to its structure [41,49]. The sorptivity of the brick samples was determined by monitoring the increase in weight of a specimen over time, during capillary water absorption. This part of the study was conducted using the direct gravimetric method, which requires samples in the form of a rectangular or cylindrical prism to ensure reproducible and comparable measurements—both of which are essential for reliable material property assessment.

The specimen must be of constant cross-sectional area parallel to the absorbing face. Before the test, measurements of all historical samples were taken to precisely determine the contact area with water. The surface area was reduced in the case of damaged corners or edges. Before testing, the samples were dried to a constant weight and placed on their sides on rods in a tray containing water (vertical capillary rise experiment), so that the entire lower surface of the specimen was in good contact with the liquid. The samples were weighed at regular intervals as the study was being conducted, to determine the quantity of liquid absorbed as described in the work of [32]. For each specimen, seven points were obtained (the specified minimum is five points). The tests were conducted for a period of approximately 1 h, up to the point where the graph of the weight gain and the square root of time showed linearity. The sorptivity is determined according to the Equation (4):

(4) $$\frac{W}{A}=S\cdot t^{\frac{1}{2}}$$

where S—is the sorptivity [mm·min^−1/2^], W—the volume of water absorbed [mm^3^], A—the sample surface area exposed to water [mm^2^], and t—the elapsed time [min].

The capillary absorption coefficient *A_cap_* and S, related to each other via the water density, serves as a quantitative descriptor of the material’s hygro-physical behavior, enabling advanced modeling of moisture transport dynamics and durability forecasting under variable environmental conditions [37]. The values of the coefficient were determined for both historic and modern types of brick according to the Equation (5):

(5) $$A_{cap}=S\cdot \rho_{w}$$

where *A_cap_*—the capillary absorption coefficient [kg·m^−2^·s^−1/2^], S—the sorptivity [mm·min^−1/2^], and *ρ*_w_—the water density [kg·m^−3^].

#### 2.2.5. Compressive Strength Tests

Compressive strength was measured according to the standard PN-EN 772-1+A1:2015-10 [50]. This parameter constitutes a critical indicator for assessing the load-bearing performance and structural stability of ceramic materials, offering quantitative insight into their degradation potential, and long-term durability under combined mechanical loading and environmental stressors. Strength tests were carried out on historical bricks as well as currently produced bricks after frost resistance testing. Due to significant irregularities, the historic brick samples were prepared by grinding the surfaces until the required flatness and parallelism were achieved. To obtain the required number of samples according to the standard and considering the condition of some of the specimen, the bricks were split in half in such a way that the height/width ratio was not less than 0.4. The samples were then conditioned to achieve an air-dry state. Constant mass was deemed to have been reached when, in subsequent weight tests (at 24 h intervals), the loss in mass between two determinations was less than 0.2% of the total mass. The conditioned samples (6 specimens of each type) were loaded in the Controls MI, Italy strength machine with a loading rate of 0.15 (N/mm^2^)/s. The compressive strength values for modern bricks before the frost resistance testing were determined in [43], while this study determined the corresponding values following frost resistance testing. This approach enabled the calculation of a strength retention ratio, serving as a quantitative indicator of the material’s resistance to freeze–thaw degradation—a key durability metric for assessing frost-induced microstructural damage and performance decline. However, compressive strength after freeze–thaw cycles was determined in this study, and the results were compared to the compressive strengths of bricks from the same series, which had not been exposed to the freeze–thaw cycles. In this way, it was possible to determine a ratio between compressive strengths—before and after the freeze–thaw cycles.

#### 2.2.6. Freezing and Thawing Resistance

The direct resistance of bricks to freeze–thaw cycles was tested according to the PN-B-12012:2022-07 standard [51], on both handmade and historical bricks, despite the latter having been previously exposed to weather conditions, including multiple freeze–thaw cycles. All the samples were collected in accordance with PN-EN 771-1+A1:2015 [52] to ensure representativity and material homogeneity crucial for reproducible results. Six selected renovation and three historical bricks of each type were subjected to the tests. The samples were saturated in water for 48 h prior to testing and placed in a climate chamber (Uni-mors, Grodzisk Mazowiecki, Poland). The temperature stability in the frost resistance test chamber is +/−1 °C, with a temperature range from −30 °C to + 30 °C. According to chosen standard, the number of freeze–thaw cycles required to assess freeze–thaw resistance is 25 cycles for ceramic products intended for use in severe conditions. In each cycle the samples were exposed to temperature of −15 ± 2 °C for four hours and then defrosted in water at a temperature of +10 °C to +25 °C. After testing, a brick sample was considered durable against freeze–thaw cycles if the number and size of edge and corner damage, as well as surface cracking, were below the limits specified in PN-B-12012:2022-07. Additionally, if none of the products subjected to cyclic freeze–thaw testing exhibited any damage, those products were deemed frost-resistant. After being exposed to the freeze–thaw cycles, compressive strength after freezing, was measured on ten brick samples, according to EN 772-1+A1:2015-10 [50].

## 3. Results and Discussion

### 3.1. Analysis of Physical and Geometric Properties

The mean dimensions of the historical bricks were 295.5–308.0 mm in length (L), 135.0–155.0 mm in width (W), and 65.0–77.5 mm in height (H). The significant dispersion in these results indicates inherent material heterogeneity and a low degree of manufacturing standardization, which was typical for production methods used from the medieval period until the 19th-century introduction of mechanized production. Although the bricks originate from the Baroque period (18th and 19th centuries), their dimensions are more characteristic of older Gothic bricks rather than the smaller “Saxon” format (approximately 250 × 133 × 45 mm) that was widely used in the 17th century [2]. While these bricks cannot be classified into standard dimensional categories, this analysis establishes the specific dimensional ranges that must be considered when selecting compatible restoration materials. Similarly, brick color was quantified using the Munsell color system to guide material selection. This approach provides objective, reproducible data reflecting surface mineralogy and firing conditions, which are critical determinants of the bricks’ physicochemical characteristics and long-term durability. The hue of the analyzed samples fell within the 5YR to 7.5YR range, indicating a transition from reddish-brown to yellowish-brown tones, with chroma values between 6 and 8. All samples were identified as reddish-brown, with variations in chroma attributed to the presence of iron oxides like hematite and magnetite. The specific dimensions and colors of both the historical and modern replacement bricks are presented in Table 1.

Table 2 presents the mean values for bulk density and water absorption by weight for both historical and modern handmade bricks, determined before and after freeze–thaw cycling. The reported values are the average of three samples tested for each brick type.

The initial water absorption of the Baroque bricks ranged from 12.7% to 14.2%, indicating a relatively consistent level of this parameter across all historical samples. These values did not correspond to those of the modern handmade samples (10.2% and 18.7%), but they were comparable to values reported for medieval bricks (13.6% to 14.5%) [43]. Similarly, the bulk density of the historical bricks was comparable to that of medieval bricks, which may indicate a continuation of local brickmaking traditions using similar raw materials and production techniques, ranging from 1770.5 to 1875.6 kg/m^3^, which is significantly higher than the density of the modern reference bricks [43]. In the next stage, tests of bulk density and water absorption were also carried out after the frost resistance tests. For the historical bricks, the assessment of water absorption was not possible due to the destruction of the samples during testing. However, it should be emphasized that, due to the lack of a clear correlation between the water absorption results prior to the freeze–thaw cycles for both historical and modern bricks, the inclusion of post-frost resistance water absorption values for both types of material would not have had a significant impact on the final conclusions of the study. The historical samples exhibited considerable variability in their physical properties. Their bulk density was comparable to that of medieval bricks [43], which may indicate a continuation of local brickmaking traditions using similar raw materials and production techniques. However, the bulk density of the historical bricks exceeded that of modern bricks. This difference in bulk density is indicative of variations in microstructural compactness and porosity distribution, which are key factors determining the material’s hygrothermal and mechanical performance.

All historical brick samples exhibited a decrease in bulk density, indicating microstructural degradation. The bulk density values were 1664.9 kg/m^3^ for M-GO and 1564.0 kg/m^3^ for M-HM with slight increase that may be related to the low density of modern bricks. An increase in density for fired bricks was also observed in [53]. Furthermore, during the freezing process, unfrozen water is gradually drawn towards ice crystallizing in larger pores (i.e., cryosuction), which generates negative water pressure in the pores that can densify the material [54]. This phenomenon highlights the complex interaction between pore structure, water phase changes, and mechanical stress, which governs freeze–thaw durability—a core focus within materials science for porous building materials.

As stated in [55], the water absorption of a brick body during freeze–thaw cycles increases linearly with the gradual change in pore structure. Similarly, water absorption test results for M-GO and M-HM bricks showed slightly increased values of 19.9% and 11.7%, respectively.

### 3.2. Mineralogical and Chemical Composition

X-ray fluorescence spectroscopy (XRF) was used to determine the chemical composition of the brick samples by quantifying their oxide content, providing essential insights into the raw material characteristics and firing conditions that directly influence microstructural development and resultant physical properties. Conversely, X-ray diffraction (XRD) was used to evaluate mineralogical composition, allowing for the identification of crystalline phases present in the tested materials. Analyses were carried out on two samples of each brick type.

XRF analysis revealed that the bricks are composed of high amounts of SiO_2_ and Fe_2_O_3_, and low amounts of flux (K_2_O, CaO). The high amount of SiO_2_ (~60%) and low amount of TiO_2_ suggest that the tested bricks were of good quality, with resistance to cracking and spalling, as TiO_2_ acts as a minor but influential phase modifier affecting thermal stability and mechanical behavior [56]. The TiO_2_ content in all the samples was similar (ranging from 2.09% to 2.37%), while the SiO_2_ content was lower in the historical brick samples (ranging from 53.36% to 60.62%), with the SiO_2_ content in modern bricks reaching 68.83% in the M-GO brick. Clay, the primary raw material in ceramic production, is generally classified by its CaO content as either calcareous (>6% CaO) or non-calcareous (<6% CaO) [57,58]. The major oxide composition showed that both historic bricks, H-SM and H-SB, as well as modern bricks, were produced from calcareous clay sources, as their CaO content was greater than 6%. In contrast, the CaO content in the H-OR and H-ML bricks was significantly lower than 6% (between 2.74–3.77%), indicating they were produced from non-calcareous clay. The difference in CaO content among the samples influenced their physical properties. The samples with higher CaO content exhibited lower porosity compared to the others. The porosity of the samples with CaO greater than 10% were measured at between 24.52% and 36.09%, whereas the samples with lower CaO content had porosity values ranging from 22.97% to 28.83%. The qualitative outcomes of the XRF analysis for all brick types are summarized in Table 3.

**Figure 2:**
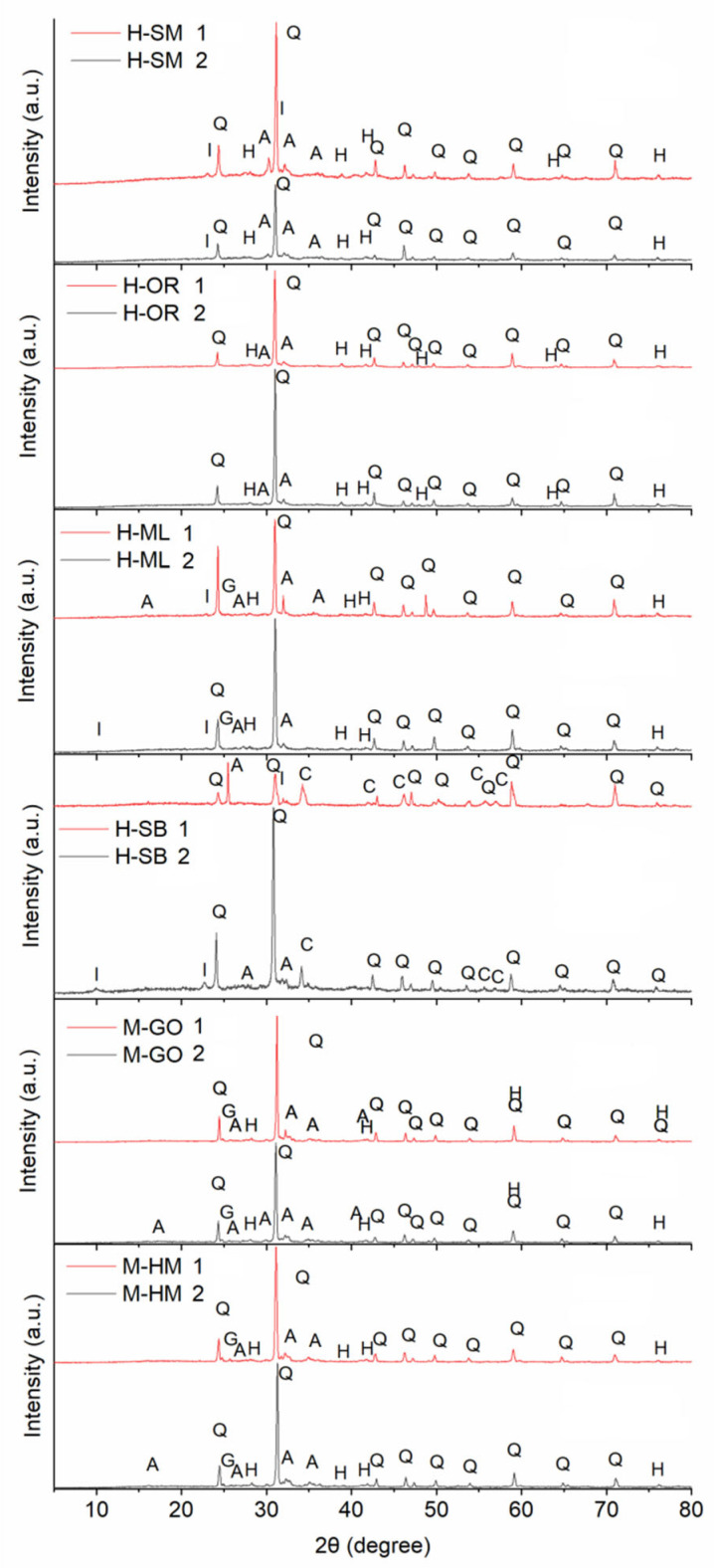
XRD patterns of historic and renovation bricks (Q: Quartz, H: Hematite, I: Illite, A: Albite, G: Geothite, C: Calcite).

The mineralogical phases determined by the use of X-ray diffraction are presented in Table 4, while the corresponding XRD patterns are presented in Figure 2. Quartz and hematite were found to be the major phases present in all brick samples. Quartz is a primary component of both the historic and restoration bricks. This result was anticipated, as quartz is a major component of all brick clays, suggesting the use of similar raw material sources and comparable manufacturing techniques. This composition differs from that of Baroque bricks in other regional studies, which report a predominance of dehydroxylated illite, quartz, and calcite, indicating the use of different argillaceous and carbonate-rich raw materials [59].

The major minerals in all samples are quartz, with proportions ranging from 42.23 to 60.35 wt%, feldspars (albite), and hematite (up to 11.54 wt%). The presence and quantity of hematite in Ca-poor bricks may indicate that the firing temperature of the bricks was approximately 850 °C, as hematite is a derivative of calcium-poor clays at high temperatures. This temperature-dependent formation of hematite significantly influences the bricks’ thermal stability and durability. In calcareous bricks, its presence may suggest that during firing, iron oxidation occurred at high temperatures, particularly in the presence of calcium, which promotes the formation of this mineral. Such mineralogical indicators are essential for reconstructing the firing regimes and predicting long-term material performance under environmental stresses.

The high concentration of goethite (up to 15.52 wt% in the H-SM sample) was unexpected, given that this iron(III) oxide-hydroxide typically transforms into hematite during high-temperature ceramic firing. Its presence, however, may be attributed to the long-term hydrothermal or acidic weathering of the brick, a process where hematite can dissolve and reprecipitate as goethite via a dissolution–precipitation mechanism [60]. The detection of calcite in the H-SB samples likely indicates secondary carbonate precipitation. Furthermore, the illite found in the H-ML and H-SB samples is probably a remnant of the original raw clay. The incomplete transformation of clay minerals during firing would also explain why aluminum oxide was either absent or present only in minimal quantities.

**Figure 3:**
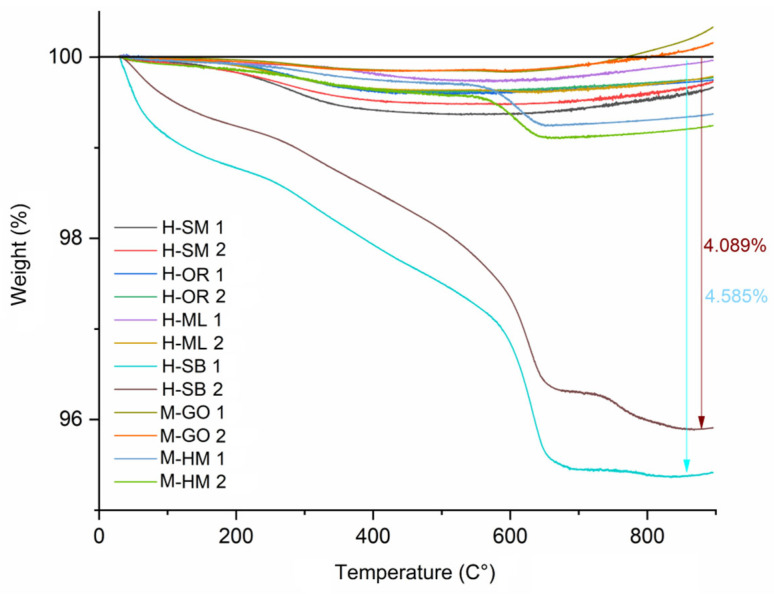
TGA graphs for historic and renovation bricks.

During the firing process of bricks, mass losses observed in the temperature range of 25–400 °C are associated with the removal of water from the brick structure. Specifically, losses between 25–100 °C correspond to the evaporation of physically adsorbed water, while those between 100–400 °C result from the dehydration of chemically bound water [60,61,62]. Mass losses occurring between 400–600 °C are primarily related to dehydroxylation processes [62,63], whereas the oxidation of organic compounds contributes to additional weight loss within the range of 200–600 °C [57,60]. The thermogravimetric analysis (TGA) results indicate distinct variations of weight loss among the analyzed samples, suggesting differences in thermal stability and decomposition mechanisms (Figure 3).

The M-GO series exhibited the lowest weight losses (ranging from 0.144% to 0.164%), indicative of superior thermal stability. In a similar manner, the H-ML and H-OR series demonstrated relatively low mass loss values, with H-ML samples exhibiting losses between 0.268% and 0.377%, and H-OR samples ranging from 0.383% to 0.401%. The M-HM and H-SM series samples demonstrated moderate yet still relatively low, weight losses of 0.758% and 0.887%, while H-SM series showed values ranging from 0.515–0.625% losses. These weight losses are most likely associated with the desorption of physically bound water and the phase transformation of goethite to hematite, a process accompanied by the release of a water molecule. Significant weight losses were recorded at approximately 700 °C for the H-OR and H-SB samples, likely resulting from the decomposition of carbonates, which generally takes place within the 700–800 °C temperature range [60,61,62]. The highest weight losses were observed in the H-SB series, which are attributed to the thermal decomposition of calcite into calcium oxide, accompanied by the release of carbon dioxide at elevated temperatures.

### 3.3. Microstructure Studies

Microstructural analysis was conducted using optical microscopy and mercury porosimetry to obtain both qualitative and quantitative insights into the pore architecture and surface morphology of historical and modern brick samples. For the microscopic examination, all historical and modern brick samples were illuminated with an artificial light source at a color temperature of 3300 K and imaged at 48× magnification.

**Figure 4:**
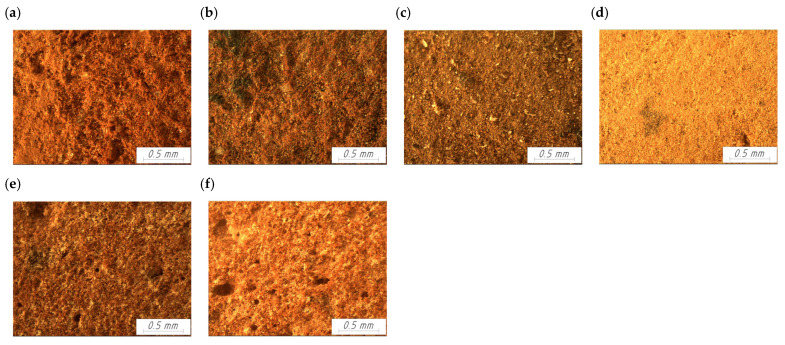
Microscopic images of the analyzed ceramic samples: (**a**) Palace in Smolajny H-SM; (**b**) Monastery in Orneta H-OR; (**c**) Church in Międzylesie, H-ML; (**d**) Synagogue in Barczewo, H-SB; (**e**) Gothic style brick M-GO; (**f**) Handmade brick M-HM.

Microscopic imaging revealed that the surfaces of the historical brick samples (Figure 4a–d) were generally less porous compared to the modern bricks (Figure 4e,f), indicating differences in raw material composition and firing regimes that influence the microstructural compactness and pore connectivity. The H-SM bricks exhibited an irregular, slightly porous surface. The H-OR bricks were characterized by a heterogeneous surface texture, with the presence of both fine and medium-sized pores. In the case of the H-ML brick, a fine-grained structure with a limited number of open pores was observed. The surface was uniform, with low visible porosity. The H-SB brick displayed a very compact and smooth surface with a minimal number of visible pores. In contrast, the modern brick samples M-GO and M-HM exhibited a relatively high number of deep pores, clearly visible in the microscopic images.

The microstructural analysis continued with mercury intrusion porosimetry (MIP) to determine key characteristics such as pore volume, surface area, and pore size distribution, in order to gain understanding of material behavior at the microscale. For each test, three specimens per brick type were analyzed across dedicated low- and high-pressure ranges. To evaluate the impact of weathering, samples were tested both before and after the freeze–thaw durability cycles.

Based on the literature defining frost-sensitive pore sizes [27,44], the pore distribution was categorized into five ranges: >10.0 µm, 10.0–3.0 µm, 3.0–1.0 µm, 1.0–0.1 µm, and <0.1 µm. This classification reflects a materials science approach to linking pore morphology with frost damage susceptibility, critical for predicting durability and failure mechanisms in porous ceramics. This approach allows for the identification of pore structures most susceptible to frost damage. To ensure the reliability of the findings and to draw well-supported conclusions, a statistical analysis was performed, allowing for the verification of observed differences and the interpretation of key material behavior patterns.

**Figure 5:**
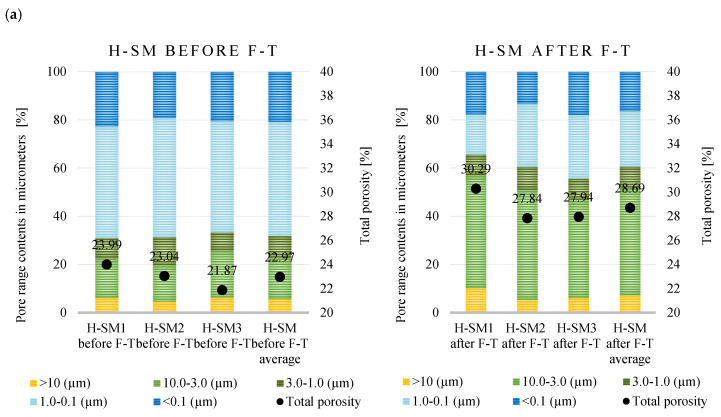

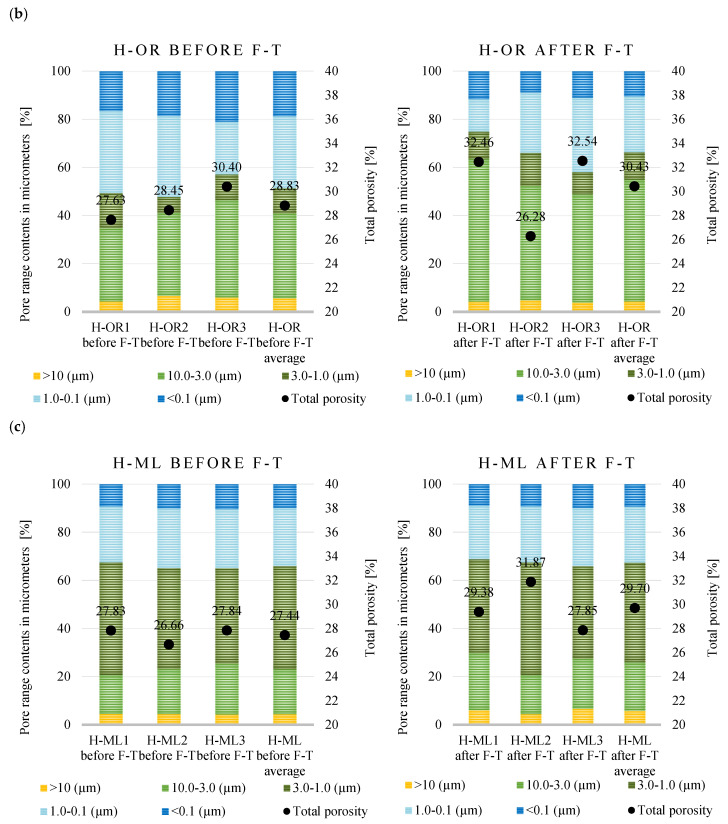

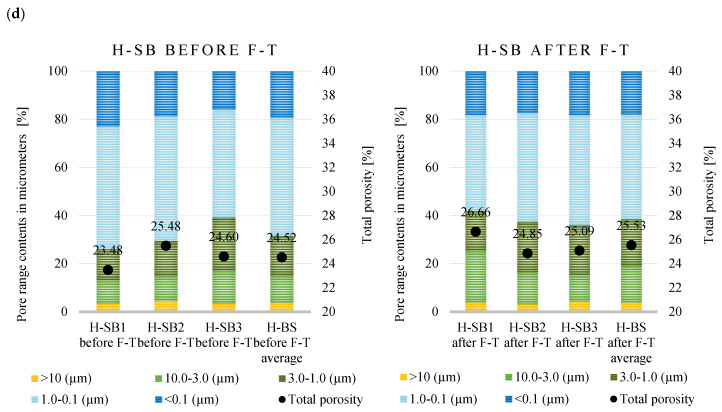
Porosity distribution in historical bricks before and after freeze–thaw testing (F–T): (**a**) Palace in Smolajny H-SM; (**b**) monastery in Orneta H-OR; (**c**) church in Międzylesie, H-ML; (**d**) synagogue in Barczewo, H-SB.

The porosity profiles determined for the historical bricks are shown in Figure 5a–d.

Analysis of the pore structure of H-SM, H-OR, H-ML, and H-SB brick samples before and after the freeze–thaw cycles showed differences in both pore size distribution and total porosity value. The H-SM series bricks were characterized by a relatively compact microstructure, and the average porosity increased from 22.97% before the F–T test to 28.69% after its completion. The pores with diameters of 0.1–1.0 µm were the main contributors to porosity structure before the F–T test, while after the test, the pores in the range of 3.0–10.0 µm predominated, indicating the formation of microcracks and pore coalescence—processes extensively described in the context of material degradation. In the H-OR samples, the highest average porosity was noted among the analyzed series before the test, amounting to 28.83%, which increased to 30.43% after the F–T cycles. The pore distribution before the test was uniform in the range of 0.1 to 1.0 µm and 3.0–10 µm, while after the tests, the pores in the range of 3.0–10 µm clearly dominated. The H-ML series showed the greatest variability in terms of total porosity, the average value of which increased from 27.44% to 32.11%, with one of the samples (H-ML2) recording a value of 39.28%. In comparison to the samples after frost resistance, a significant decrease in the pore share was observed in the range < 0.1 µm. The H-SB series bricks showed the smallest differences in total porosity, with an increase from 24.52% to 25.53% after F–T testing. The pore distribution in this series remained relatively stable, with a dominant share of pores with diameters ranging from 0.1 to 1.0 µm both before and after the freeze–thaw cycles. Table 5 summarizes key microstructural parameters for the historical bricks: total porosity, total pore area, and the average pore diameter (4V/A). These metrics are essential in materials science, linking microstructural features with functional properties; total porosity reflects the void fraction affecting mechanical strength and permeability, total pore area relates to reactive surface influencing durability, and average pore diameter serves as an indicator of pore connectivity and potential microcrack presence. Total porosity indicates the volume fraction of voids; a higher value generally corresponds to a less dense microstructure. The total pore area represents the internal surface area accessible to mercury, where a high value suggests significant surface activity and an abundance of fine pores. The average pore diameter (4V/A) is calculated as four times the ratio of total pore volume to total surface area and provides an effective diameter assuming a simplified cylindrical pore model. An increase in this parameter suggests the formation of larger pores or microcracks, which directly impacts the material’s permeability and susceptibility to degradation.

The first two groups of ceramic samples, marked with the H-SB symbol from Palace in Smolajny and H-OR from the monastery in Orneta, exhibit a similar nature of microstructural changes. In both cases, after the F–T cycles, an increase in total porosity is observed, a decrease in the total pore area (H-SM: from 2.41 to 1.12 m^2^/g; H-OR: from 3.05 to 1.63 m^2^/g) and an increase in the average pore diameter (H-SM: from 0.20 to 0.57 µm; H-OR: from 0.21 to 0.47 µm). These transformations are indicative of pore coalescence and microcrack initiation and propagation mechanisms driven by cyclic freeze–thaw loading. Such microstructural evolutions critically govern key functional properties of the material, including permeability, fracture resistance, and long-term structural stability under fluctuating environmental and mechanical stressors. The group of bricks H-ML from the church in Miedzylesie are characterized by a different course of changes. In their case, after the F–T test, a significant increase in total porosity and a significant decrease in pore surface area (from 4.66 to 0.55 m^2^/g) were noted, with a simultaneous rapid increase in the average pore diameter (from 0.13 to 1.53 µm). In turn, the H-SB bricks (from the synagogue in Barczewo) show a different trend, characterized by a stable microstructure. After the F–T test, the values of all three parameters remain practically unchanged: total porosity increased slightly by about one percentage point, the pore surface area remained at a high level (from 6.70 to 5.92 m^2^/g), and the average pore diameter remained almost constant (increase of 0.01 µm). This means no significant changes in the pore structure. To assess the impact of freeze–thaw (F–T) cycles on the porosity of historical bricks, a paired t-test was applied to the collected data. In the H-SM group, a statistically significant increase in porosity was observed: t = −12.254, *p* = 0.007. In the remaining groups—H-OR, H-ML, and H-BS—the changes in porosity after F–T cycles were not statistically significant (*p* = 0.507, 0.229, and 0.465, respectively), although an upward trend in mean values was noted. These results suggest varied microstructural resistance among the tested materials, with only the H-SM bricks showing statistically confirmed degradation following exposure to F–T cycles.

**Figure 6:**
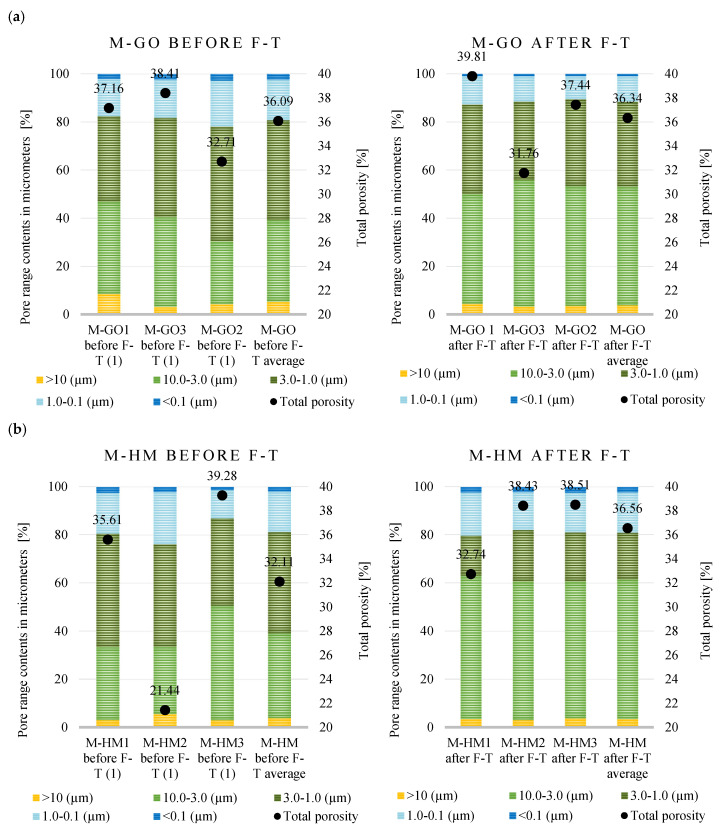
Porosity distribution before and after freeze–thaw testing (F–T) of (**a**) M-HM bricks, (**b**) M-GO bricks. (1) The values of the modern brick properties determined in [43].

The modern brick samples also underwent porosimetry analysis both before and after the freeze–thaw tests (Figure 6). The initial pre-test porosity results for these materials (M-GO before F–T and M-HM before F–T) were previously reported in [43].

Modern bricks of the M-GO series are characterized by high and uniform total porosity, which before the freeze–thaw cycles ranged from 36.09 ± 2.99%, and after the test increased slightly, reaching a maximum of 36.34 ± 4.13. The pore size distribution remains stable—pores in the range of 0.1 to 3.0 µm dominate, while before the test after the F–T tests, pores in the range of 3.0 to 10.0 µm dominate. In the case of M-HM bricks, the total porosity before the F–T test was more diverse and ranged from 21.44% to 39.28%. After the F–T cycles, pores in the range of 3.0–10.0 µm definitely began to dominate, constituting almost 60% of the total porosity.

The porosimetry results for the modern bricks, conducted before and after freeze–thaw testing, are summarized in Table 6. In the samples of modern M-GO bricks after freeze–thaw cycles, the total porosity generally did not increase and was at a level of about 36%. The values of the total pore area before and after the F–T test were 0.65 ± 0.03 m^2^/g and 0.43 ± 0.06 m^2^/g, respectively, while the average pore diameter reached 3.66 ± 0.35 µm and 3.80 ± 0.28 µm, which indicates the predominance of pores with larger diameters. For M-HM bricks, the total porosity before the F–T test was on average 32.11% (±9.42), while after the F–T cycles it increased to 36.56% (±3.30). At the same time, significant changes were noted in other parameters: the pore surface decreased from 0.55 ± 0.14 m^2^/g to 0.30 ± 0.13 m^2^/g, and the average pore diameter increased from 1.53 ± 0.30 µm to 6.66 ± 2.57 µm. Such a clear shift towards larger pore diameters, with a simultaneous decrease in surface development, indicates a significant transformation of the microstructure as a result of frost cycles.

To evaluate the impact of freeze–thaw (F–T) cycles on the porosity of contemporary bricks, a statistical analysis was conducted using the paired *t*-test. In the M-GO group, porosity increased slightly, and this change was not statistically significant (t = −0.070, *p* = 0.950). In the M-HM group, porosity rose, also without statistical significance (t = −0.707, *p* = 0.553). The results indicate no clear effect of F–T cycles on the porosity of contemporary bricks in either of the analyzed cases.

**Figure 7:**
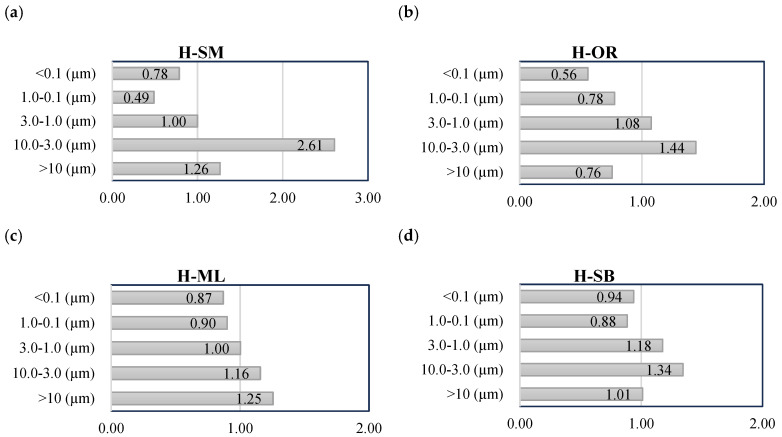
Porosity transformation coefficients for historical bricks (**a**) Palace in Smolajny H-SM; (**b**) monastery in Orneta H-OR; (**c**) church in Międzylesie, H-ML; (**d**) synagogue in Barczewo, H-SB.

In order to gain a better understanding of the microstructural mechanisms underlying frost damage, changes in pore volume within specific size ranges were analyzed for the tested historical bricks. These changes are represented by porosity transformation coefficient PTC (displayed on the *x*-axis in Figure 7), defined as the ratio of pore volume in a given size range after the frost resistance test to the initial pore volume in the same range. A coefficient greater than 1.0 indicates an increase in pore volume within that range, whereas a value below 1.0 indicates a decrease. According to Figure 7, a trend can be observed: in almost all cases, the volume of pores with diameters ranging from 10.0 to 3.0 µm increased, while the volume of pores smaller than 1.0 µm decreased. In historical bricks of the H-SM type, porosity in the 0.1–1.0 µm range decreased by more than 50%, whereas in the H-OR, H-ML, and H-SB samples, the decrease ranged from 22% to 10%. This pattern reflects the partial collapse and coalescence of finer pores into larger voids driven by internal ice crystallization pressure and cyclic freeze–thaw stresses. This may indicate partial destruction of the finest pores, which were likely transformed into larger units due to the increase in internal ice pressure and the stresses generated during the freezing of water within the brick structure. In contrast, in pores with diameters between 3.0 µm and 10.0 µm, porosity increased by 116% (H-ML) to 261% (H-SM), suggesting the formation of larger voids as a result of smaller pores merging. The freeze–thaw cycling process leads to the transformation of initially fine, non-harmful pores within the brick structure into larger porosity units, which promote the initiation of microcracks and, consequently, contribute to the reduction of the masonry element’s durability. This shift in pore distribution directly contributes to the deterioration of the material’s mechanical properties, particularly in the historical bricks, which exhibited a high structural change coefficient.

The results of the Student’s t-test for the range of 10.0–3.0 µm indicate that for all tested bricks the changes in the porosity transformation coefficient (PTC) were statistically significant at the level of *p* < 0.01. Such uniform significance of changes indicates that the range of 10.0–3.0 µm changes the most as a result of frost degradation.

### 3.4. Hygric Properties Characterization

Sorptivity studies using the gravimetric method are simple and therefore widely used in moisture determination. This method enables the quantitative assessment of water uptake kinetics, which is intrinsically linked to the pore structure and surface chemistry of the brick materials. Most old bricks have suction rate values in the range of 0.5–3.0 mm·min^−1/2^ [21,34]. Table 7 shows the results of sorptivity tests for samples of each type of brick (historic and currently produced) and the capillary absorption coefficient determined according to Equation (5).

A comparison of sorptivity values revealed a significant difference between the historical bricks from this study and the modern bricks reported in [43]. On average, the Baroque bricks exhibited sorptivity values that were approximately half those of their modern counterparts, indicating a significantly lower water absorption capacity and suggesting that modern handmade bricks may be unsuitable as replacement materials due to the critical role of capillarity in water transport within masonry. Among the historical samples, the H-SM brick had the lowest sorptivity, indicating a reduced capacity for water uptake. When contextualized with data from machine-made bricks from the early 20th century with a sorptivity of 2.2351 $mm\cdot {min}^{\frac{-1}{2}}$, as stated in [44], and the medieval samples with results ranging from 0.3252 to 0.8864 $mm\cdot {min}^{\frac{-1}{2}}$, according to the previous study of the authors [43], there is considerable variation in sorptivity across historic bricks from different periods. This underscores the importance of evaluating the water transport characteristics of bricks on a period-specific basis. The considerable variation in sorptivity reflects the temporal evolution of microstructural characteristics. Such variability highlights the critical importance of conducting period-specific assessments of hygric transport properties, particularly when selecting compatible restoration materials to ensure long-term durability and optimal performance.

As indicated in the literature [64], sorption values should reflect the dominance of a specific pore size range in modern bricks. Furthermore, publication [16] suggests that the relationship between the water absorption coefficient and porosity can be nearly linear.

**Figure 8:**
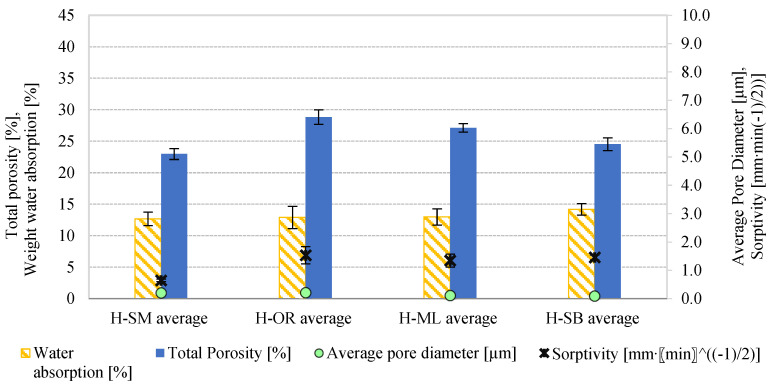
The comparison of results for total porosity [%], average pore diameter [µm], weight water absorption [%], and sorptivity [$mm\cdot {min}^{\frac{-1}{2}}$] for historical bricks.

Figure 8 shows the relationship between total porosity, average pore diameter, sorptivity, and water absorption for four types of historical bricks (H-SM, H-OR, H-ML, and H-SB), with error bars included to represent the standard deviation of the measured values. The highest sorptivity (1.5316 $mm\cdot {min}^{\frac{-1}{2}}$) was observed in the H-OR sample, which had the highest porosity (28.83%) and relatively large pores (0.21 µm). As also noted in study [16], sorptivity tends to increase with porosity. The H-SB brick, despite having lower porosity (24.52%) and the smallest pores (0.09 µm), also showed high sorptivity (1.4584 $mm\cdot {min}^{\frac{-1}{2}}$). The lowest value was recorded for H-SM (0.6472 $mm\cdot {min}^{\frac{-1}{2}}$), which had the largest pores and the lowest porosity. A similar trend was observed for water absorption—the highest was for H-SB (14.19%) and the lowest for H-SM (12.68%). These findings confirm that both porosity and pore size significantly influence the moisture behavior of the material.

**Figure 9:**
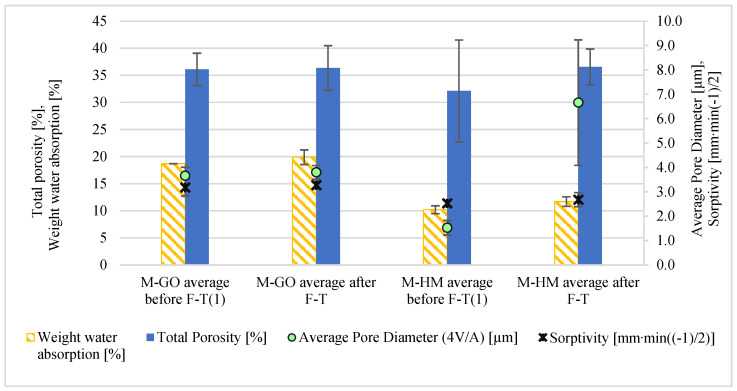
The comparison of results for total porosity [%], average pore diameter [µm], weight water absorption [%], and sorptivity [$mm\cdot {min}^{\frac{-1}{2}}$] for modern bricks before and after F–T. (1) The value of Total porosity of the modern brick properties determined in [43].

The analysis of the data for modern bricks, presented in Figure 9, indicates that after F–T cycles the observed increase in total porosity leads to higher values of average pore diameter, sorptivity, and water absorption, which is consistent with the observations given in [16]. In both samples of modern bricks (M-HM and M-GO), the trend is the same.

### 3.5. Compressive Strength Tests

Compressive strength measurements for historical (18th and 19th century) and contemporary bricks are summarized in Table 8.

The mean compressive strength for the historical bricks was calculated to be 13.49 $N/{mm}^{2}$, while the range is dispersed in the range of 7.72–22.11 $N/{mm}^{2}$. However, the results are similar to those obtained in other studies on bricks from analogous time periods [21,22,65,66]. The mean compressive strength for handmade samples before exposure to freeze/thaw cycles was calculated to be 18.23 $N/{mm}^{2}$, while the range was 17.61–18.85 $N/{mm}^{2}$ [43], whereas after exposure to freeze/thaw cycles, it was calculated to be 14.83 $N/{mm}^{2}$, and the range was 14.27–15.39 $N/{mm}^{2}$, indicating consistent compressive strength values both before and after frost resistance testing. The decrease in strength results from the impact of microstructural changes induced by cyclic freezing and thawing on the material’s mechanical integrity. The results of pre- and post-freeze–thaw cycle compressive strengths of currently produced bricks coincide with the middle of the range, in reference to the literature. For comparison, similar historical bricks have compressive strengths ranging from 5 to 20 MPa, with their relatively low strength generally attributed to the presence of large pores and microcracks in the material structure [66,67].

**Figure 10:**
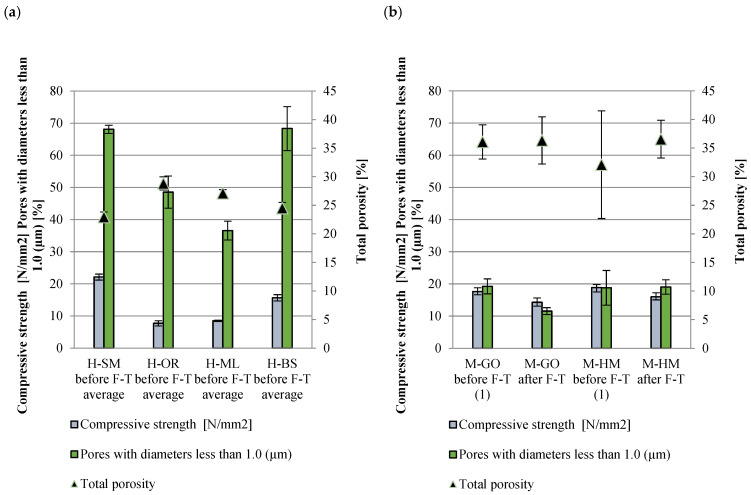
Porosity distribution with respect to ranges influencing compressive strength (**a**) for historical bricks and (**b**) for modern handmade bricks. (1) The values of the modern brick properties determined in [43].

The comparative analysis of historical and modern bricks (H-SM, H-OR, H-ML, H-BS, M-HM, and M-GO), presented in Figure 10, indicates correlations between compressive strength, total porosity, and the proportion of pores with a diameter below 1.0 µm. To highlight the variability of the data, error bars representing standard deviation were included.

The mechanical strength of the bricks is intrinsically linked to their porosity and pore structure. An optimal distribution, characterized by a high proportion of fine pores and low total porosity, correlates with higher strength. Conversely, unfavorable changes, such as a reduction in fine pores combined with increased total porosity, lead to the degradation of the material’s mechanical properties. Historical bricks, such as H-SM and H-SB, are characterized by high compressive strength and a significant proportion of pores with a diameter below 1.0 µm (68.1% and 68.3%). At the same time, their total porosity remains relatively low (22.97% and 24.52%), which has a positive effect on mechanical durability. In comparison, modern bricks (M-GO and M-HM) show a clear decrease in strength after freeze–thaw cycles. This deterioration is accompanied by a reduction in the proportion of pores with a diameter below 1.0 µm (e.g., in M-GO from 19.2% to 11.5%) and an increase in total porosity.

### 3.6. Assessment of Frost Resistance

**Figure 11:**
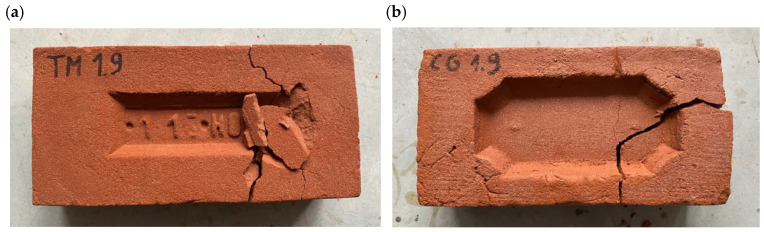
Non-resistant bricks after the freeze–thaw study: (**a**) handmade building brick M-HM (**b**) gothic style brick M-GO.

According to the criteria specified by the standard PN-B-12012:2022-07, if at least one of the products shows any damage, they can be considered non-resistant to freeze–thaw cycles within the conditions of use for which they were assessed. As it is shown in Figure 11 both types of handmade bricks exposed to twenty-five freeze–thaw cycles were damaged during the study. Most of the tested samples exhibited damage in the form of deep cracks, which consequently caused the brick to break into smaller pieces. No damage in the form of delaminations or edge and angle damage were observed. Frost resistance tests were also conducted on historical bricks, which were subjected to twenty-five freeze–thaw cycles (three samples of each type). All tested samples were damaged. However, in contrast to the contemporary bricks, the damage appeared as delamination or fine cracks. From a materials science standpoint, these damage patterns exemplify fundamental microstructural degradation mechanisms induced by cyclic freeze–thaw loading. The quantified increase in total porosity and average pore diameter post freeze–thaw cycling substantiates matrix disruption, evidencing microcrack nucleation and pore coalescence phenomena that critically undermine the structural integrity of the material. All tested bricks demonstrated a statistically significant increase in both total porosity and average pore diameter following freeze–thaw cycling, providing quantitative evidence of microstructural degradation. These results underscore the pivotal role of pore morphology evolution in governing frost-induced damage mechanisms and subsequent mechanical property deterioration.

The compressive strengths of the bricks before and after freeze–thaw cycles, with the corresponding standard deviation and ratio between the pre- and post-freeze–thaw cycle compressive strengths, are shown in Table 9. They were calculated using the average values of compressive strength before and after freeze–thaw cycles.

According to [68] the compressive strength ratio pre- to post-freezing is a good classifier in the sense that all non-resistant bricks have a ratio between 0.68 and 0.72, and resistant bricks have a higher ratio between 0.72 and 0.89. The results obtained for both contemporary brick samples would suggest their resistance to frost damage; however, the conducted tests revealed that they do not meet the requirements for frost resistance. Based on the partial data, the Student’s t-test for dependent samples was performed to assess the effect of freeze–thaw (F–T) cycles on the compressive strength of modern M-GO and M-HM ceramic bricks. Following freeze–thaw cycling, the compressive strength of M-GO samples decreased by 3.34 N/mm^2^, while M-HM samples exhibited a reduction of 2.86 N/mm^2^. In both cases, the observed differences were statistically significant (M-GO: t = 4.38, *p* = 0.048; M-HM: t = 10.74, *p* = 0.009). These results demonstrate that exposure to freeze–thaw cycles induces a significant deterioration in compressive strength, underscoring the susceptibility of modern ceramic bricks to degradation under cyclic frost conditions, as confirmed by statistical analysis. In order to better understand the impact of water on the course of freeze–thaw cycles, and the associated structural degradation, it would be advisable to introduce advanced methods enabling the distinction between free, bound, and structural water within the porous structure of ceramic materials, in accordance with the publications [69,70].

## 4. Conclusions

This study investigates the physical, hygric, and mechanical properties of Baroque ceramic bricks originating from heritage structures in northeastern Poland. A comparative analysis was conducted to assess the compatibility of modern hand-made bricks with historical materials, with an emphasis on a materials science perspective. The methodology included porosity profiling via mercury intrusion porosimetry, freeze–thaw resistance testing, compressive strength measurements, and statistical evaluation of the results. The following conclusions are drawn:

A Freeze–thaw cycles led to a statistically significant reduction in compressive strength in both modern brick types (M-GO and M-HM), confirming their vulnerability to frost-induced mechanical degradation.
B The pore size range of 10.0–3.0 µm was identified as particularly sensitive to freeze–thaw damage, indicating its relevance as a key durability indicator for ceramic building materials exposed to cyclic thermal stress. This range exhibited consistent changes across tested bricks, supported by statistical significance (*p* < 0.01).
C Despite systematic comparative analysis, no consistent relationship was found between the physical, hygric, and mechanical parameters of historical and modern bricks. This highlights the high heterogeneity of historical ceramic materials and underscores the limitations of direct substitution with contemporary analogues.
D The results provide new experimental data on pore structure and the moisture-related behavior of heritage bricks, which may serve as input parameters for numerical modeling of hygrothermal performance in historic masonry structures.
E Future studies should incorporate a broader sample pool and apply advanced material characterization methods, such as SEM and micro-CT, to better understand complex degradation mechanisms—particularly those beyond freeze–thaw action, including salt crystallization, chemical weathering, and biological colonization.

In conclusion, this study advances the materials science-based understanding of historical ceramic bricks by identifying their microstructural vulnerabilities to freeze–thaw degradation, while also underscoring the need for further research to evaluate their performance under diverse environmental and mechanical stressors.

## Figures and Tables

**Table 1:** Dimension and color of bricks determined using a spectrophotometer.

Sample	Dimensions			Color-Code	Color Name	Color
	L [mm]	W [mm]	H [mm]			
H-SM	308.0	145.0	65.0	7.5YR 3/8	Dark strong reddish brown	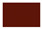
H-OR	300.5	146.5	74.5	7.5YR 5/6	Reddish brown	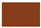
H-ML	308.0	155.0	77.5	5YR 4/6	Moderate to strong reddish brown	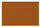
H-SB	295.5	135.0	68.0	5YR 5/6	Reddish brown	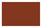
M-GO	280.0	140.0	85.0	7.5YR 4/8	Strong reddish brown	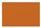
M-HM	250.0	120.0	65.0	7.5YR 4/6	Moderate reddish brown	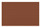

**Table 2:** Physical properties of historic and restoration bricks pre- and post freeze–thaw cycles with corresponding standard deviation.

Samples	Bulk Density Before F–T Cycles [kg/m^3^]	Bulk Density After F–T Cycles [kg/m^3^]	Water Absorption Before F–T Cycles [% m/m]	Water Absorption After F–T Cycles [% m/m]
H-SM	1900.7 ± 21.64	1829.0 ± 4.77	12.7 ± 1.07	-
H-OR	1800.5 ± 52.22	1717.7 ± 50.73	12.9 ± 1.76	-
H-ML	1845.4 ± 1.86	1818.8 ± 27.00	13.0 ± 1.29	-
H-SB	1860.80 ± 27.37	1685.1 ± 130.85	14.2 ± 0.90	-
M-GO	1521.0 ± 2.38 (1)	1664.9 ± 2.24	18.7 ± 0.12 (1)	19.9 ± 1.35
M-HM	1518.0 ± 2.26 (1)	1564.0 ± 2.47	10.2 ± 0.72 (1)	11.7 ± 0.88

(1) The values of the modern brick properties determined in [43].

**Table 3:** Chemical composition of historic and renovation bricks determined by XRF.

Brick Type	% SiO_2_	%Fe_2_O_3_	%K_2_O	% CaO	% TiO_2_	%ZrO_2_	MnO
H-SM	53.76	29.79	7.27	6.25	2.09	0.36	0.30
H-OR	55.89	30.92	7.11	2.74	2.24	0.41	0.25
H-ML	60.62	24.04	8.33	3.77	2.24	0.37	0.25
H-SB	53.36	25.98	6.89	10.42	2.37	0.39	0.30
M-GO	68.83	11.46	3.61	11.53	2.16	0.53	0.24
M-HM	65.25	13.09	4.87	13.02	2.19	0.95	0.27

**Table 4:** Mineralogical composition of bricks assessed by X-ray diffraction analysis (XRD).

Brick Type	Quartz (w/w%)	Hematite (w/w%)	Illite (w/w%)	Albite (w/w%)	Geotite (w/w%)	Calcite (w/w%)	Amorphous (w/w%)
H-SM	44.78	4.11	-	10.04	15.52	-	28.10
H-OR	48.49	11.54	-	21.00	-	-	18.95
H-ML	44.80	3.85	17.59	11.71	1.88	-	20.15
H-SB	42.23	-	11.45	14.2	-	7.65	25.73
M-GO	60.35	4.35	-	17.10	2.95	-	15.25
M-HM	59.15	4.75	-	16.85	2.90	-	16.31

**Table 5:** Summary of the microstructure features of historical bricks before and after the freeze–thaw study with corresponding standard deviation.

Brick Type	Total Porosity [%]	Total Pore Area [m^2^/g]	Average Pore Diameter (4V/A) [µm]	t (Paired)	*p*-Value
H-SM before F–T	22.97 ± 0.87	2.41 ± 0.11	0.20 ± 0.02	−12.254	0.007
H-SM after F–T	28.69 ± 1.13	1.12 ± 0.17	0.57 ± 0.06	-	-
H-OR before F–T	28.83 ± 1.16	3.05 ± 0.29	0.21 ± 0.02	−0.802	0.507
H-OR after F–T	30.43 ± 3.59	1.63 ± 0.70	0.47 ± 0.14	-	-
H-ML before F–T	27.13 ± 0.62	5.21 ± 0.52	0.11 ± 0.01	−1.713	0.229
H-ML after F–T	29,70 ± 2.03	4.81 ± 0.20	0.13 ± 0.03	-	-
H-SB before F–T	24.52 ± 1.00	6.70 ± 0.43	0.08 ± 0.01	−0.896	0.465
H-SB after F–T	25.53 ± 0.98	5.92 ± 0.42	0.09 ± 0.01	-	-

**Table 6:** Summary of the microstructure features of modern bricks before and after the freeze–thaw study with corresponding standard deviation.

Brick Type	Total Porosity [%]	Total Pore Area [m^2^/g]	Average Pore Diameter (4V/A) [µm]	t (Paired)	*p*-Value
M-GO before F–T	36.09 ± 2.99 (1)	0.65 ± 0.03	3.66 ± 0.35	−0.707	0.95
M-GO after F–T	36.34 ± 4.13	0.43 ± 0.06	3.80 ± 0.28	-	-
M-HM before F–T	32.11 ± 9.42 (1)	0.55 ± 0.14	1.53 ± 0.30	−0.07	0.553
M-HM after F–T	36.56 ± 3.30	0.30 ± 0.03	6.66 ± 2.57	-	-

(1) The values of the modern brick properties determined in [43].

**Table 7:** Sorptivity and capillary absorption coefficient results for brick samples with the corresponding standard deviation.

Brick Type	Sorptivity $\;[mm\cdot {min}^{\frac{-1}{2}}]$	Capillary Absorption Coefficient $\;[kg\cdot m^{-2}\cdot s^{\frac{-1}{2}}]$
H-SM	0.647 ± 0.043	0.083
H-OR	1.532 ± 0.303	0.197
H-ML	1.347 ± 0.230	0.174
H-SB	1.458 ± 0.084	0.188
M-GO before F–T	3.1807 ± 0.351 (1)	0.410
M-HM before F–T	2.5354 ± 0.146 (1)	0.327
M-GO after F–T	3.276 ± 0.121	0.422
M-HM after F–T	2.688 ± 0.289	0.346

(1) The values of the modern brick properties determined in [43].

**Table 8:** Compressive strength graph with corresponding standard deviation values.

Brick Type	Compressive Strength [N/mm^2^]
H-SM	22.11 ± 0.94
H-OR	7.72 ± 0.82
H-ML	8.49 ± 0.24
H-BS	15.66 ± 0.98
M-GO before F–T (1)	17.61 ± 1.18
M-GO after F–T	14.27 ± 1.35
M-HM before F–T (1)	18.85 ± 0.94
M-HM after F–T	15.99 ± 1.25

(1) The values of the modern brick properties determined in [43].

**Table 9:** Compressive strength values of modern bricks before and after freezing with the corresponding standard deviation and the ratio of compressive strengths pre- to post-freezing.

Brick Type/Property	Gothic Style Brick (M-GO)	Handmade Building Brick (M-HM)
Compressive strength before exposure to freeze/thaw cycles (N/mm^2^)	17.61 ± 2.36 (1)	18.85 ± 1.88 (1)
Compressive strength after exposure to freeze/thaw cycles (N/mm^2^)	14.27 ± 1.39	15.39 ± 1.06
Ratio of compressive strength after and before exposure to freeze/thaw cycles	0.81	0.82
t (paired)/*p*-value	4.38/0.048	10.74/0.009

(1) The values of the modern brick properties determined in [43].

## Data Availability

The original contributions presented in the study are included in the article, further inquiries can be directe d to the corresponding author.
